# Delayed mating in the malaria vector *Anopheles funestus* compared to *Anopheles arabiensis* (Diptera: Culicidae)

**DOI:** 10.1093/jme/tjaf059

**Published:** 2025-05-08

**Authors:** Emmanuel Elirehema Hape, Alex Thadei Ngonyani, Daniel Mathias Mabula, Joel Daniel Nkya, Claus Augustino Thomas, Mohamed Jumanne Omari, Doreen Josen Siria, Halfan Said Ngowo, Lizette Leonie Koekemoer, Fredros Oketch Okumu

**Affiliations:** Environmental Health and Ecological Science Department, Ifakara Health Institute, Ifakara, Tanzania; Wits Research Institute for Malaria, Faculty of Health Sciences, University of the Witwatersrand, Johannesburg, South Africa; Environmental Health and Ecological Science Department, Ifakara Health Institute, Ifakara, Tanzania; Environmental Health and Ecological Science Department, Ifakara Health Institute, Ifakara, Tanzania; Environmental Health and Ecological Science Department, Ifakara Health Institute, Ifakara, Tanzania; Institute of Biodiversity, Animal Health, and Comparative Medicine, G12 8QQ, University of Glasgow, Glasgow, UK; Department of Microbiology and Parasitology, St. Francis University College of Health and Allied Sciences (SFUCHAS), Ifakara, Tanzania; Environmental Health and Ecological Science Department, Ifakara Health Institute, Ifakara, Tanzania; Institute of Biodiversity, Animal Health, and Comparative Medicine, G12 8QQ, University of Glasgow, Glasgow, UK; Environmental Health and Ecological Science Department, Ifakara Health Institute, Ifakara, Tanzania; Institute of Biodiversity, Animal Health, and Comparative Medicine, G12 8QQ, University of Glasgow, Glasgow, UK; Environmental Health and Ecological Science Department, Ifakara Health Institute, Ifakara, Tanzania; Wits Research Institute for Malaria, Faculty of Health Sciences, University of the Witwatersrand, Johannesburg, South Africa; Vector Control Reference Laboratory, Centre for Emerging Zoonotic and Parasitic Diseases, National Institute for Communicable Diseases, A Division of the National Health Laboratory Services, Johannesburg, South Africa; Environmental Health and Ecological Science Department, Ifakara Health Institute, Ifakara, Tanzania; Wits Research Institute for Malaria, Faculty of Health Sciences, University of the Witwatersrand, Johannesburg, South Africa; Institute of Biodiversity, Animal Health, and Comparative Medicine, G12 8QQ, University of Glasgow, Glasgow, UK

**Keywords:** insemination, reproductive behavior, light wavelength, malaria vectors

## Abstract

Mating is a vital behavior for mosquito reproduction, yet it remains poorly understood under captive conditions. We examined the copulation dynamics of 2 key malaria vectors, *Anopheles funestus sensu stricto* Giles and *Anopheles arabiensis* Patton, under laboratory settings in Tanzania. We conducted observations in 24-h cycles and monitored copulation events and insemination of females, initially using flashlights for nighttime visibility, followed by red lights in subsequent experiments. We observed how variations in mosquito age and artificial lighting influenced mating success for these 2 mosquito species within cages under controlled conditions. We found that *An. arabiensis* copulated relatively soon after emergence, with 32.4% of individuals mating by day 3 postemergence, whereas *An. funestus* showed delayed activity, reaching a similar mating frequency by day 8. The introduction of artificial red light significantly accelerated copulation in *An. funestus* but did not affect *An. arabiensis*. Sperm transfer and mating plug delivery in over 92% of copulating pairs of both species was confirmed by dissection. Mating occurred primarily at night, with distinct peaks at 10 PM for *An. arabiensis* and 11 PM for *An. funestus*. In conclusion, our findings revealed species-specific differences in reproductive behavior, which could improve the colonization of *An. funestus*, a species historically challenging to rear in captivity. These insights also may facilitate the development of new vector control technologies, such as sterile insect techniques and genetic-based approaches, that exploit mosquito mating behavior.

## Introduction

Vector control methods, particularly using insecticide-treated nets (ITNs) and indoor residual spraying (IRS), remain the cornerstone of malaria control in Africa, but are increasingly challenged by adaptive mosquito behaviors and widespread insecticide resistance (Bhatt et al. 2015, Sanou et al. 2021, Odero et al. 2024). Although much is known about the biting and feeding patterns of *Anopheles* mosquitoes, critical gaps remain in understanding their copulation and mating behaviors, particularly for those of key vectors such as *Anopheles funestus sensu stricto* Giles and *Anopheles arabiensis* Patton (Carrasco et al. 2019, Takken et al. 2024).

Mating behavior of *Anopheles*, including swarming and copulation (genitalia engagement), is influenced by environmental cues such as light, temperature, and humidity (McIver 1980, Howell and Knols 2009). However, the specifics of these behaviors and their variation among species remain poorly understood, particularly under natural and seminatural conditions (Paton et al. 2013, Facchinelli et al. 2015). Light intensity plays a crucial role in the onset and timing of mating (coupling: male seizing female), yet its effects remain underexplored. This study examines how lighting influences mating activity in *An. funestus* and *An. arabiensis*, key malaria vectors with different colonization challenges. Although *An. arabiensis* readily adapts to laboratory conditions, *An. funestus* presents significant challenges for colony maintenance, with only a few strains, such as FUMOZ from Mozambique in 2000, FANG from Angola in 2002 (Hunt et al. 2005), and recently FUTAZ from Tanzania, being successfully maintained for extended generations (Ngowo et al. 2021). Previous studies suggest that low light can trigger male swarming (McIver 1980, Gary et al. 2009, Sawadogo et al. 2014, Kaindoa et al. 2017, 2019, Charlwood 2023), but species-specific responses and their implications for vector control strategies remain unclear. Understanding how light influences mating behavior could provide valuable insights for optimizing colony maintenance and advancing malaria control efforts (Coetzee and Fontenille 2004, Nepomichene et al. 2017, Ngowo et al. 2021, Kahamba et al. 2022).

Understanding copulation dynamics is essential for developing innovative vector control strategies, such as the sterile insect technique (SIT) and gene drives (Benelli and Beier 2017). These methods rely heavily on manipulating reproductive success, with SIT focusing on releasing sterile males to compete with wild males for wild females and gene drives aiming to propagate specific genetic traits that suppress populations or block disease transmission (Helinski et al. 2008, Dame et al. 2009). The success of such approaches depends on understanding factors that influence mating success, including male competitiveness, female choice, and the impact of environmental cues (Mclver 1980, Howell and Knols 2009). In areas where multiple *Anopheles* species coexist, interspecific mating and potential hybridization could further complicate control efforts, particularly if genes conferring insecticide resistance or other adaptive traits are transferred (Slotman et al. 2004, Choochote et al. 2014, Niang et al. 2015).

Behavioral flexibility among *Anopheles* species also contributes to residual malaria transmission. For example, *An. arabiensis* frequently rests outdoors and exhibits opportunistic feeding on humans and animals, evading traditional indoor-based interventions such as ITNs and IRS (Carrasco et al. 2019). These behaviors, coupled with the species’ mating strategies, necessitate a broader focus on their reproductive ecology. Similarly, *An. coluzzii*, which thrives in urban environments, demonstrates ecological adaptability that supports its role as a significant malaria vector in densely populated regions (Niang et al. 2015, Kamau et al. 2024).

Studies under controlled conditions have begun to shed light on the mating systems of these vectors, revealing the importance of population density, sex ratios, and environmental factors in determining mating outcomes (Facchinelli et al. 2015). Such research highlights the importance of targeting reproductive behavior to enhance vector control. For example, SIT programs must ensure that sterile males effectively compete for mates, whereas gene drives require a comprehensive understanding of mating networks to achieve efficient dissemination of modified traits (Dame et al. 2009). Moreover, exploring factors such as light intensity and wavelength, which influence swarming and copulation activities, could inform the design of interventions that disrupt mating success in natural settings (Rowland 1989, Gibson 1995, Sheppard et al. 2017, Hellhammer et al. 2022, Finda et al. 2023).

Given the challenges posed by insecticide resistance and behavioral adaptations, integrating reproductive behavior research into malaria control programs offers a promising avenue for reducing mosquito populations and disrupting transmission. By advancing our understanding of *Anopheles* mating dynamics, particularly for less-studied species like *An. funestus* and *An. arabiensis*, targeted and sustainable strategies can be developed to combat malaria in regions like Tanzania and beyond. In the current study, we investigated the copulation dynamics of *An. funestus* and *An. arabiensis* in the laboratory, with a primary focus on how mosquito age and light intensity influence mating success.

## Material and Methods

### Mosquitoes

The experiments used (i) *An. arabiensis* colonized since 2009 (Batista et al. 2019), (ii) *An. funestus* FUTAZ colonized since 2020, and (iii) *An. funestus* FUMOZ colonized since 2018 at Ifakara (originally in 2000 in South Africa). Mosquitoes used in the experiments were reared under insectary conditions with no windows, maintained at 27 ± 2 °C, at the 12:12 L:D photoperiod, and housed in a 30 × 30 cm caging system. Long-term colonization may enhance mating within cages, potentially influencing the behaviors we observed.

### Laboratory Observations of the Effects of Mosquito Age on Copulation Events in *An. funestus* and *An. arabiensis*

We first investigated the copulation dynamics of *An. arabiensis* and both FUMOZ and FUTAZ strains of *An. funestus*. This study involved 24-h observations, with 3 replicates per species and strain, inside 30 × 30 cm cages containing approximately 1,500 mosquitoes in a sex ratio of 1 female for every 2 males. Observations were made continuously for 16 consecutive days by 3 trained observers working in 8-h shifts, counting the number of copulations visually without camera assistance. The first set of experimental observations was done following the 12:12 L:D photoperiod, whereby the day-time period was initiated by turning on the light (36 W, AC220 to 240 V, 50/60 Hz) at 7 AM and a nighttime period initiated by turning off the light at 6 PM. The room (no windows) did not allow for gradual sunset and sunrise conditions. In the first set of experiments, mosquito mating, which typically occurs midair in nature, in a caging system, the clasped pairs usually descend to the cage floor, making a hit sound that signals copulation’s start. Observers used a hand-held flashlight (1 W high power LED; HL-558) to count events until mating stopped, then turned it off, awaiting resumption, as detection in total darkness was not feasible.

The first set of experiments used a flashlight for nighttime observations, whereas the second set replaced it with a 7 W red-light bulb to determine whether the previous light source influenced the mating observations. Each condition used separate mosquito batches rather than simultaneous controls. The red light followed the same 12:12 L:D photoperiod, turning on at 6 PM and off at 6 AM. The red light was chosen because it closely mimics the wavelengths present during sunset, which were less likely to disrupt mosquito behavior (Sheppard et al. 2017, Hellhammer et al. 2022). Specifically, the Eurolux G434RDL Red B22 230V 7 W Globe LED used typically emits light in the 620 to 630 nm range, a part of the spectrum where mosquito visual sensitivity is reduced, minimizing potential impact on mating behavior (Sheppard et al. 2017). No additional flashlight was used under red-light conditions, relying solely on the 7 W red-light bulb (Eurolux G434RDL Red B22 230V 7 W Globe LED, Lumen 150 lm) to observe copulation activities.

A HOBO Temp/RH/Light/Ext-Analog Data Logger^@^ recorded light intensity (LUX) continuously, with laboratory conditions ranging from near zero lux at “night” to 80 lux during “day” (flashlight) or 25 lux (red light), compared to field conditions peaking at 55,000 lux. The natural light environment in the field was measured as a control, by placing a similar HOBO logger under a tree. However, only light levels were recorded without observing swarming activities, previously reported to occur around 6:40 PM for approximately 12 (±5 SD) min (Kaindoa et al. 2017, 2019).

### Laboratory Confirmation of the Successful Insemination and Transfer of Mating Plugs to the Females Captured *in copula*, and of the Sexual Maturity of the Copulating Males

**Fig. 1.**
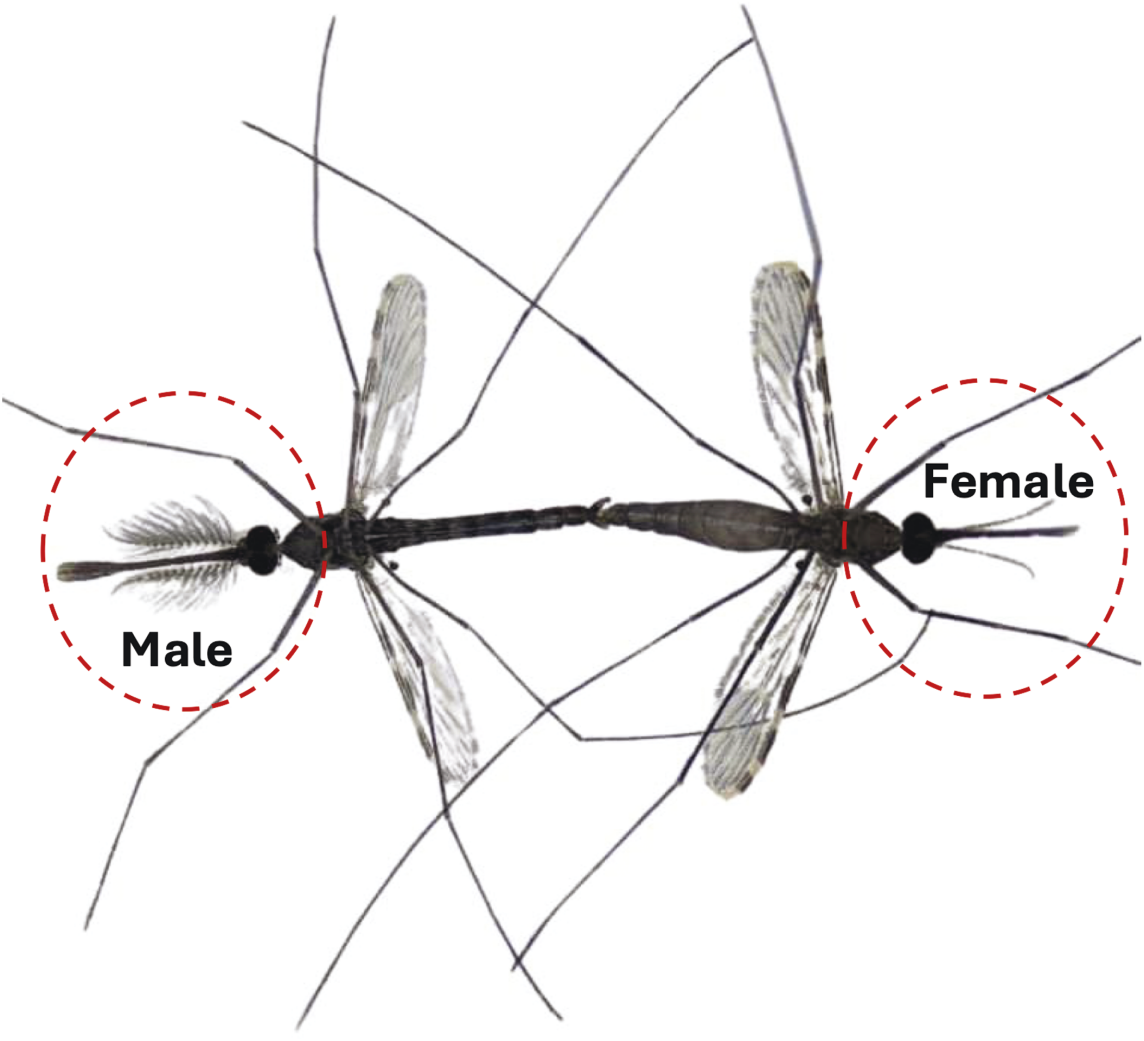
Male–female copulation: a male and female *Anopheles* mosquito in copula, demonstrating the typical mating posture where the male clasps the female during swarming behavior.

To confirm mating success, successful insemination and transfer of mating plugs, and male sexual maturity, a random number of intact copulating pairs (5 to 10 per cage per day during mating periods) were retrieved from the cage within 2 to 5 s using a mouth aspirator and killed by freezing for 10 min (Fig. 1). Observations were conducted without the red light, and hand-held flashlights were used to assist in the procedure. Mating success was confirmed by dissecting the females to assess insemination. To do this, the terminalia and last abdominal segment (segment IX) were cut open in saline to expose the spermatheca capsule. Slide mounts of spermatheca capsules were inspected using a compound microscope at 10× magnification to confirm the presence of sperm (Ngowo et al. 2021). A stereo microscope was used to assess the transfer of the mating plug and the completion of the 180° rotation of male genitalia *in copula* (Oliva et al. 2011).

### Laboratory Analysis of Peak Mating Times in Mosquitoes at Optimal Mating Age

Adult mosquitoes reared under controlled laboratory conditions were observed continuously to study their peak mating activities over a 24-h cycle. In the first experiments using flashlights, mosquitoes were first aged to Day 3 (*An. arabiensis*) or Day 8 (*An. funestus* FUTAZ) and 10 (*An. funestus* FUMOZ) that were identified as the optimal ages for maximum copulation, based on the observations described above. Three replicates of 1,500 mosquitoes (500 females, 1,000 males) per cage were conducted for each strain, using one batch for flashlight experiments. Swarming and copulation activities in captivity were monitored continuously, starting at 6 AM and extending through the night and following day, completing a full 24-h observation period.

### Statistical Analysis

Data were statistically analyed using R-software Version 4.4.1 to investigate the impact of mosquito age (in days), light type, and light intensity on the copulation of different *Anopheles* strains (*An. arabiensis*, FUTAZ, and FUMOZ). Generalized Linear Mixed Models (GLMMs), using the R package, “*lme4*” (Bates et al. 2015) were used to estimate the mosquito age (in days) at which the maximum number of copulations were recorded as well as the effects of light-type on copulation. The number of copulations observed was modeled following a “*Poisson*” distribution with mosquito strain and light type as fixed effects. Experimental replicates were included as random effects in each of the models generated. Additionally, the proportions of female mosquitoes inseminated, having mating plugs, and males with completed 180° of genitalia rotation retrieved in copula were expressed as mean percentages. The peak time of copulation during the 24-h observation was calculated as the mean number of copulations per hour. The 24-h light intensity (LUX) was calculated using the 16-d mean average of the readings obtained using the HOBO Data Logger^@^ in the laboratory and the field environment. Model selection was done by progressively deleting terms from the maximal model with the “*drop1()*” function, and likelihood ratio tests were employed to assess the relevance of explanatory variables. The “*ggplot2*”, R programmes were used to create all the graphics (Wickham 2016).

## Results

### Mating Age and Peak Copulations

**Fig. 2.**
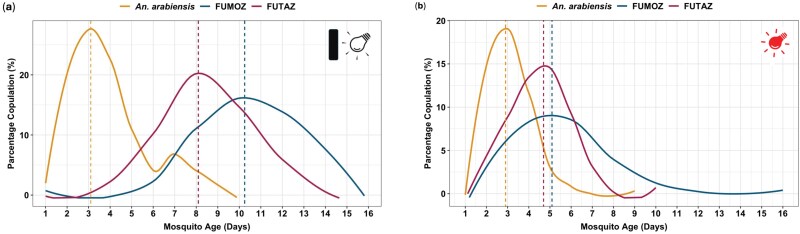
Smoothed percentage of copulation events per day across 3 replicates (*geom_smooth*), representing daily totals, not continuous observations. A) Copulation events recorded under dark conditions using a flashlight for counting. B) Copulation events recorded under a 7-W red light, allowing observation and counting without a flashlight. The vertical dashed lines indicate the age for maximum copulation activity for the specific species and strains.

For all *Anopheles* strains, the percent of total of copulation events per day showed a curvilinear relationship with mosquito age (days since emergence). Under conditions without supplementary night light (Fig. 2A), *An. arabiensis* reached its copulation frequency peak by day 3 postemergence, accounting for 32.4% of total observed copulations. In contrast, *An. funestus* colonies experienced significant delays, with FUTAZ peaking at day 8 (26.3% of total copulations) and FUMOZ at day 10 (37.7% of copulations).

**Fig. 3.**
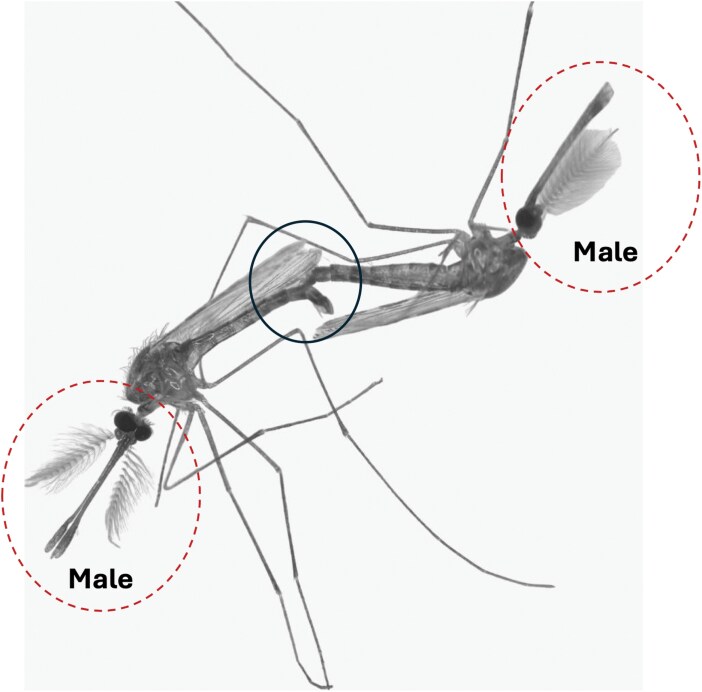
Male–male copulation: a rare occurrence, 2 male *Anopheles* mosquitoes are seen in copula. Unlike typical copulation, one male grabbed the abdomen of the other male rather than the last abdominal segment (black circle). The behavior often resulting from mistaken identity or high competition, highlights the complexity of mosquito mating dynamics under laboratory conditions.

The use of red light in the insectary did not significantly impact the copulation duration of *An. arabiensis*. However, light type significantly reduced the age at which peak copulation activity was observed (*χ*^2^ = 7,582.2, df = 4, *P* < 0.001), with peak copulation occurring at ~5 d postemergence (*χ*^2^ = 4,952.3, df = 3, *P* < 0.001) in both the FUMOZ and FUTAZ strains. Compared to conditions without red light, strain-specific differences were also significant (*χ*^2^ = 853.04, df = 2, *P* < 0.001; Fig. 2B). On rare occasions, male–male copulation was observed in *An. funestus* (Fig. 3).

Cumulative mating frequency over a 3-d window around the peak were calculated: *An. arabiensis* achieved ~70% mating at days 2 to 4 under flashlight conditions, while *An. funestus* (FUTAZ) reached ~60% over days 7 to 9, and FUMOZ ~75% over days 9 to 11. Under red light, FUTAZ and FUMOZ peaked at day 5, with cumulative rates of ~65% and ~80% over days 4 to 6, respectively.

#### Timing of Copulation and Mating

**Fig. 4.**
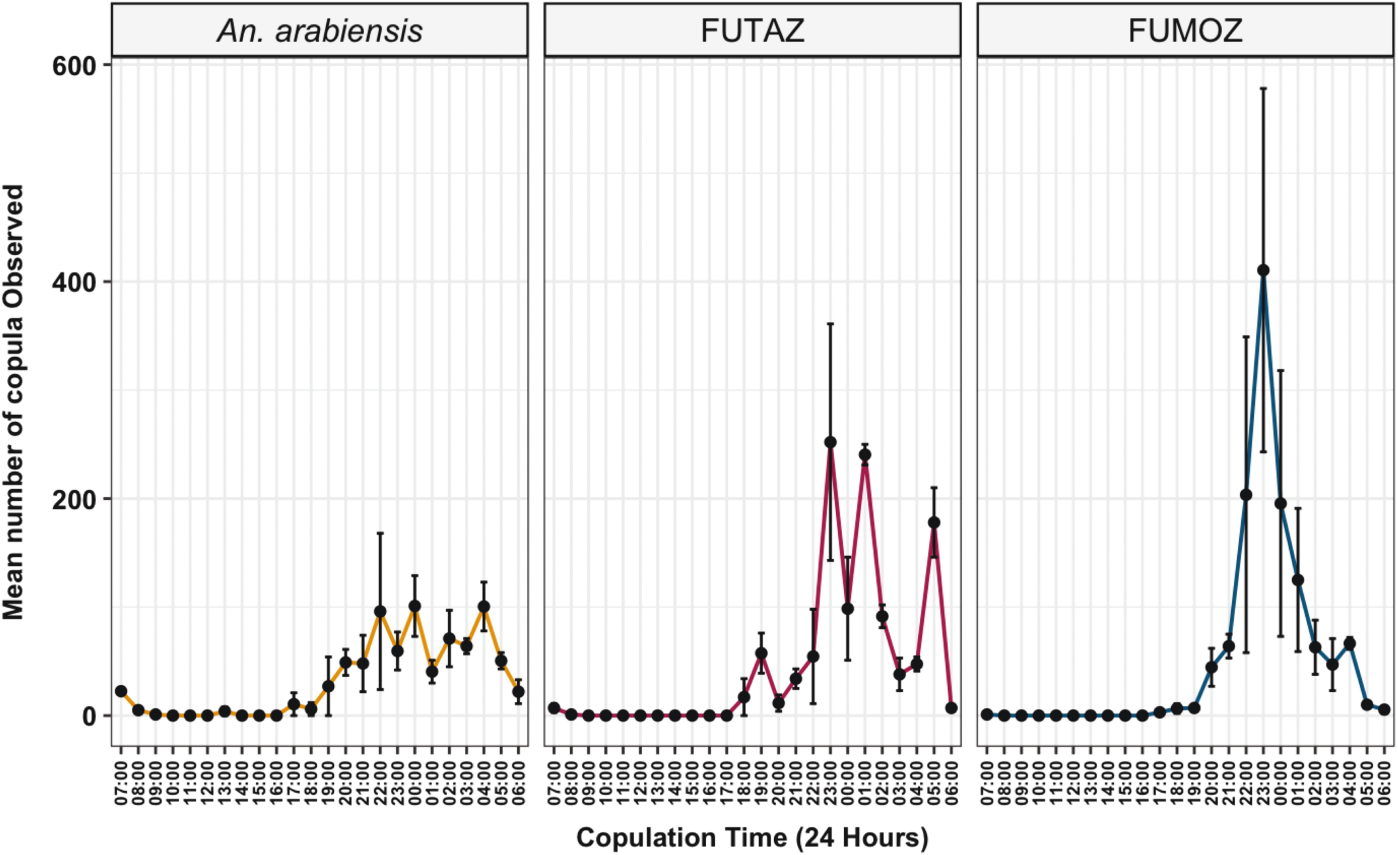
Mean number of copulations per hour from 3 replicate cages, with error bars representing the standard deviation during the age when each species reached their maximum mating activity.

During the night of maximum copulation activity (Fig. 2), copulation activity persisted throughout the light-off period, although there were differences in peak timing among the species and strains. For *An. arabiensis*, mating was initiated as early as 5 PM (before light off) and continued until around 6 AM, occasionally extending to 7 AM (after light on). The FUTAZ strain of *An. funestus*, however, demonstrated early mating initiation at 6 PM, with erratic activity throughout the night, persisting until 6 AM the following morning (Fig. 4). In contrast, *An. funestus* (FUMOZ strain) showed a later onset of mating, commencing around 8 PM and reaching peak activity by 11 PM before concluding by 5 AM.

#### Successful Insemination of Females and Sexual Maturity of the Males

**Fig. 5.**
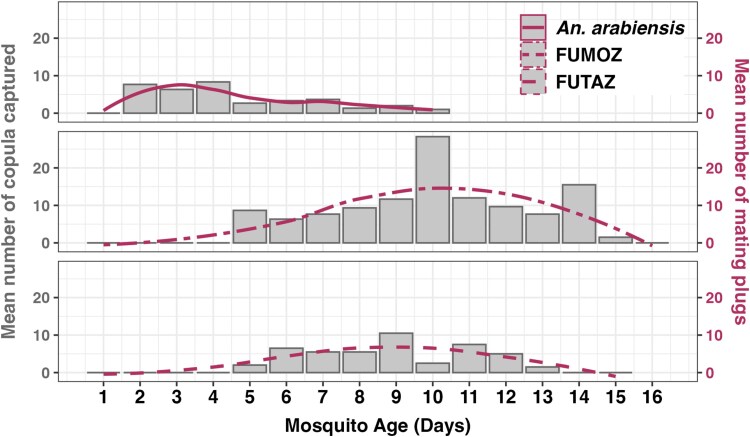
Mean number of *An. arabiensis* and *An. funestus* (FUMOZ and FUTAZ) collected in copula per day and the number of females containing mating plugs. Over 92% of the randomly collected females from mating pairs from different age groups successfully contained mating plugs. Observations were conducted using a flashlight during total darkness.

More than 92% of dissected female mosquitoes, caught while physically coupled (intact copulas), had evidence of successful transfer of sperm to the female spermathecae, along with effective delivery of mating plugs. Moreover, 96% of the male mosquitoes had completed the 180° rotation of their genitalia at the time the mating pairs were captured, indicating sexual maturity (Table 1). The number of copulation events was directly related to successful mating events (as indicated by the transfer of mating plugs), and both measures exhibited a curvilinear relationship with mosquito age postemergence (Fig. 5).

**Table 1.** Number of intact copulating pairs collected during the experiment highlighting the reproductive outcomes across species

	Total no. of copula collected	% Females inseminated	% Females with mating plugs	% Males sexually matured
*An. arabiensis*	105	98.95	97.14	100.00
*An. funestus* (FUMOZ strain)	338	93.79	92.01	96.75
*An. funestus* (FUTAZ strain)	93	97.85	96.77	98.92

### Light Intensity

**Fig. 6.**
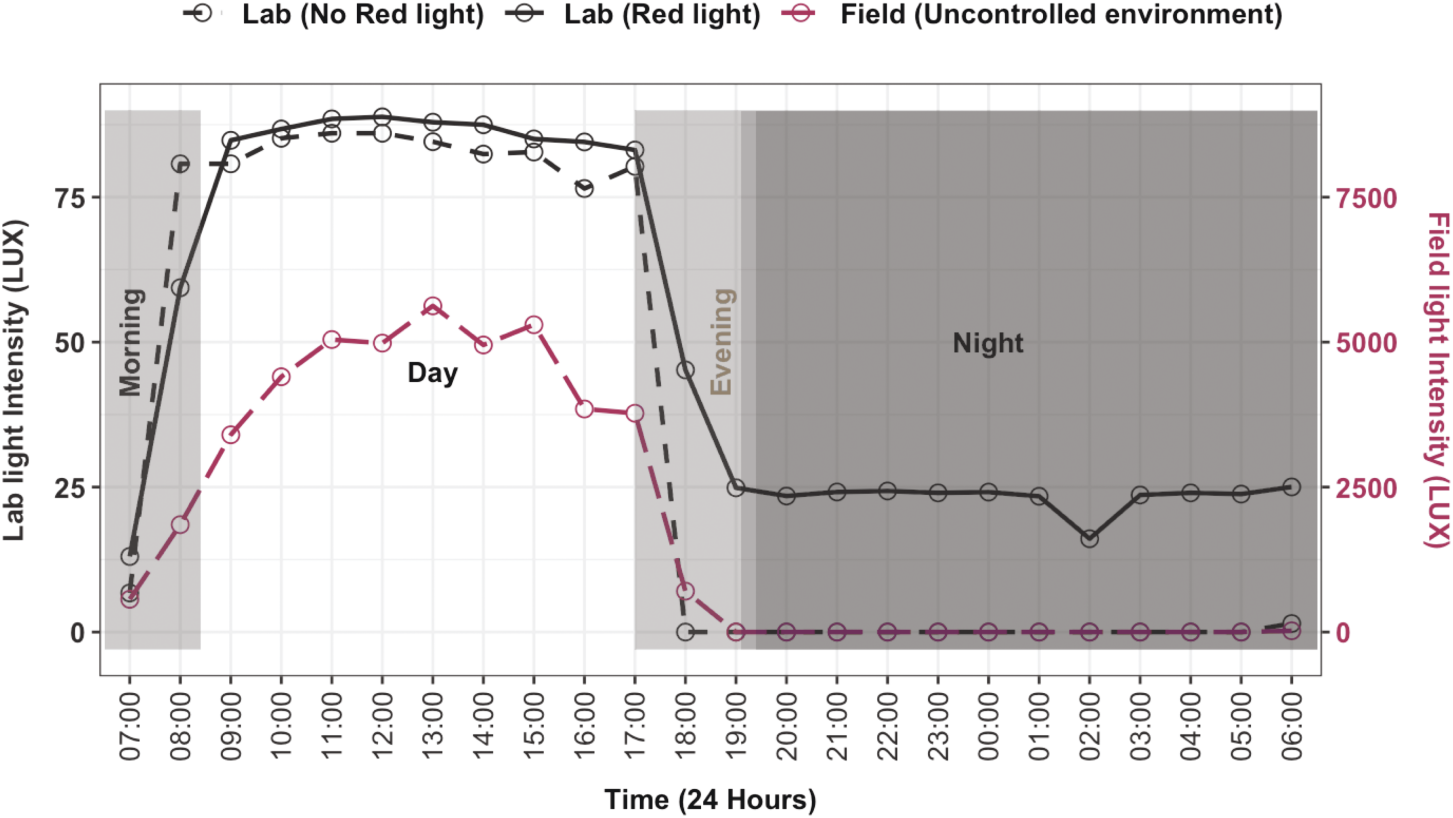
Mean light intensity over a 24-h period for “no red light”, “red light”, and “field” conditions. In laboratory settings, light switches are abrupt; the apparent gradual transition in “red light” is an artifact of averaging multiple days’ data. The “field” condition reflects natural, gradual light changes. During light-off periods, “no red light” uses intermittent flashlight illumination, while “red light” maintains constant dim lighting. Day lighting is identical across laboratory conditions.

Light intensity in the laboratory ranged from near 0 to 80 lux (flashlight) or 25 lux (red light), contrasting with field peaks of 55,000 lux (see Methods) (Fig. 6).

## Discussion

Understanding the reproductive behaviors of malaria vectors such as *An. arabiensis* and *An. funestus* is essential for tracking population dynamics and developing effective vector control strategies. Mating behaviors, including swarming and copulation, are central to mosquito reproduction, yet they remain understudied, particularly for species such as *An. funestus* that are challenging to colonize (Dia et al. 2013, Nepomichene et al. 2017, Kahamba et al. 2022). Swarming and copulation behaviors, influenced by environmental cues such as light intensity, temperature, and humidity, play a pivotal role in mosquito reproduction (Charlwood et al. 2003, Sawadogo et al. 2014, Kaindoa et al. 2019, Baeshen 2022). Our study provided a detailed comparative analysis of the copulation dynamics of 2 key malaria vectors, *An. funestus* and *An. arabiensis*, examining the number of copulation events and successful insemination, mating plugs, and genitalia rotation in retrieved copulas. These observations offer new insights into the reproductive behaviors of these vector species.

In nature, male mosquitoes form swarms at specific times and locations, guided by environmental cues such as light, temperature, and humidity. These swarms form in locations where females are likely to encounter them for mating, with competition and choice determining reproductive success (Charlwood et al. 2003, Sawadogo et al. 2014, Kaindoa et al. 2019, Baeshen 2022). Although natural swarming is limited to brief dusk periods in the field (Reisen et al. 1977), our laboratory observations here revealed prolonged mating activities throughout the night, initiating as early as 5 PM and extending to 7 AM the following morning. The peak copulation periods varied slightly between *An. arabiensis* and *An. funestus*, reflecting species-specific differences. Recent field studies also support this extended mating window, with insemination rates increasing overnight, reaching ~80% to 90% by early morning (Nambunga et al. 2021). These findings challenge the assumption of strictly time-bound swarming and mating behaviors, underscoring the importance of reconsidering temporal aspects of mosquito reproduction for control strategies.

The chronological age of mosquitoes was strongly associated with copulation events and mating dynamics across strains. Perhaps the most important finding of this study was that *An. funestus*, which, though poorly studied due to lack of laboratory colonies in many research groups, has significant delays in peak mating. Laboratory colonized *An. arabiensis* initiated copulation earlier postemergence compared to *An. funestus* strains (FUTAZ and FUMOZ), reflecting species-specific differences during this period. Previous studies on *An. gambiae* and *An. stephensi* similarly reported increases in copulation success within a few days postemergence (Suleman 1990, Oliva et al. 2011). For *An. funestus*, copulation events peaked later, likely influenced by physiological processes occurring during the precopulation period. Postpeak declines in mating activities were observed, possibly due to the experimental sex ratio (1 female: 2 males) and the reproductive principle that females typically mate only once (Benedict and Rafferty 2002, Adams and Roux 2024). These findings emphasize the importance of tailoring experimental conditions to accurately reflect natural reproductive behaviors and species-specific copulation dynamics.

Light intensity was a critical factor influencing copulation dynamics. Under total darkness, observations were conducted using a flashlight for visibility, with *An. arabiensis* peaking in mating activity at 3 d postemergence, while *An. funestus* strains FUTAZ and FUMOZ required 8 to 10 d. This supported previous studies that showed *An. funestus* optimal mating success is 8 d postemergence (Maharaj et al. 2022). However, introducing a 7-W red light significantly reduced the time to peak mating for *An. funestus*, achieving maximum copulation around 5 d. This indicated that dim red light, perhaps simulating natural dusk conditions, likely optimized reproductive behaviors. Studies on other *Anopheles* species, such as *An. gambiae*, similarly highlights low light as a trigger for swarming and mating, whereas excessive artificial light disrupts these behaviors (Rowland 1989, Vielma et al. 2023, Takken et al. 2024). The association between light intensity and mating success highlights its potential for improving laboratory colonization and production for genetic control.

In rare observations, male–male copulation was noted in *An. funestus*. Although uncommon, such behavior likely results from sensory errors, where males misidentify fellow males as mates due to movement or wingbeat frequency (Mclver 1980, Baeshen 2022) or perhaps crowded cage conditions. This behavior may also occur because males copulate rapidly to maximize their chances of siring the next generation, leading to the clasping of other males in the swarm (Howell and Knols 2009). High competition, limited female availability, and altered environmental cues in laboratory settings may exacerbate these errors (Howell and Knols 2009). While not adaptive, these instances emphasize the complexity of mating dynamics and the influence of experimental conditions on observed behaviors.

Although our study was broadly successful, there were some minor limitations. Most importantly, the artificial laboratory conditions used in this study, including fixed light intensity and sex ratios, may limit the generalizability of the findings to wild populations. Additionally, the lack of a simultaneous control group comparing flashlight and red-light conditions across the same mosquito batch limits direct attribution of differences to light type alone, as batch effects may have contributed to the observed differences. Natural variables such as fluctuating light, temperature, and humidity, as well as unexamined functions of swarming, such as navigation or predator avoidance, remain unexplored. These potential hidden functions could provide further insights into mosquito biology and vulnerabilities for vector control. For species such as *An. funestus*, which are difficult to colonize, optimizing laboratory conditions, including light intensity and mating environments, is essential for colony maintenance and advancing interventions.

## Conclusion

Our study provides new information on the mating behaviors of the 2 malaria vectors, *An. arabiensis* and *An. funestus*, providing valuable insights into their reproductive dynamics under controlled laboratory conditions. Our findings suggest that mating in these species can extend beyond the dusk period, with artificial light during the night potentially having an important impact on mating behavior. The study highlights species-specific differences in reproductive timing, which could have significant implications for both wild population dynamics and laboratory-based vector control efforts. Broadly, the study showed that mating in *An. funestus* peaks much later (nearly 5 d later) than in *An. arabiensis*. Furthermore, optimizing light conditions may improve the efficiency of colonizing *An. funestus*, a species notoriously difficult to maintain under laboratory conditions. Future research should focus on replicating these findings in field settings, exploring the role of additional environmental factors, and further elucidating the mechanisms behind species-specific reproductive behaviors for more targeted malaria vector control strategies.

## Data Availability

All data generated from this study will be available from the corresponding author as per request.
